# Spatial distribution of 12 class B notifiable infectious diseases in China: A retrospective study

**DOI:** 10.1371/journal.pone.0195568

**Published:** 2018-04-05

**Authors:** Bin Zhu, Yang Fu, Jinlin Liu, Ying Mao

**Affiliations:** 1 School of Public Policy and Administration, Xi’an Jiaotong University, Xi’an, Shaanxi, China; 2 Department of Public Policy, City University of Hong Kong, Hong Kong, China; The University of Hong Kong, CHINA

## Abstract

**Background:**

China is the largest developing country with a relatively developed public health system. To further prevent and eliminate the spread of infectious diseases, China has listed 39 notifiable infectious diseases characterized by wide prevalence or great harm, and classified them into classes A, B, and C, with severity decreasing across classes. Class A diseases have been almost eradicated in China, thus making class B diseases a priority in infectious disease prevention and control. In this retrospective study, we analyze the spatial distribution patterns of 12 class B notifiable infectious diseases that remain active all over China.

**Methods:**

Global and local Moran’s I and corresponding graphic tools are adopted to explore and visualize the global and local spatial distribution of the incidence of the selected epidemics, respectively. Inter-correlations of clustering patterns of each pair of diseases and a cumulative summary of the high/low cluster frequency of the provincial units are also provided by means of figures and maps.

**Results:**

Of the 12 most commonly notifiable class B infectious diseases, viral hepatitis and tuberculosis show high incidence rates and account for more than half of the reported cases. Almost all the diseases, except pertussis, exhibit positive spatial autocorrelation at the provincial level. All diseases feature varying spatial concentrations. Nevertheless, associations exist between spatial distribution patterns, with some provincial units displaying the same type of cluster features for two or more infectious diseases. Overall, high–low (unit with high incidence surrounded by units with high incidence, the same below) and high–high spatial cluster areas tend to be prevalent in the provincial units located in western and southwest China, whereas low–low and low–high spatial cluster areas abound in provincial units in north and east China.

**Conclusion:**

Despite the various distribution patterns of 12 class B notifiable infectious diseases, certain similarities between their spatial distributions are present. Substantial evidence is available to support disease-specific, location-specific, and disease-combined interventions. Regarding provinces that show high–high/high–low patterns of multiple diseases, comprehensive interventions targeting different diseases should be established. As to the adjacent provincial units revealing similar patterns, coordinated actions need to be taken across borders.

## Introduction

Since ancient times, humans have been battling against infectious diseases. Throughout history, various infections emerged endlessly, causing tremendous tragedy across the globe [1]. The damage caused by infectious diseases are not limited to numerous casualties and economic losses. At social level, infectious diseases disproportionately affect the population and considerably aggregate health inequities, with high economic burden and poor treatment conditions for socially disadvantaged groups [2–4]. In addition, given the huge medical expenses as a result of infectious diseases, the poorest population easily get trapped in a vicious cycle of poverty and illness [5].

To better prepare for and respond to the outbreaks of infectious diseases in the 21st century, the United Nations has positioned infectious diseases as a key part in the Millennium Development Goals (MDGs) in 2000, aiming to provoke worldwide actions in infectious disease prevention and control [6,7]. Owing to increased political commitment, rapid growth of investment, and advances in diagnostic technology and treatment, MDGs ended in 2015 and remarkable progress had been achieved in the fight against epidemics [5]. World Health Organization (WHO) reported that the number and percentage of deaths as a result of infectious diseases decreased during the MDG era [8]. Nevertheless, despite the substantial development achieved by a series of interventions, infectious diseases remain a global concern. Certain traditional infectious diseases, such as sexually transmitted infections and viral hepatitis, continue to take a heavy toll on economies and health systems [9]. In addition, a much broader spectrum of infectious disease outbreaks, such as the emerging Ebola and influenza, are challenging the emergency response capacity of all nations, particularly the developing countries.

As the largest developing country in the world, China has been threatened by infectious diseases, and the difficulty in controlling these diseases is exacerbated by rapid urbanization and population mobility in recent years. To better monitor and control the spread of infectious diseases, China enacted the *Law of the People’s Republic of China on the Prevention and Treatment of Infectious Diseases* in 1989, which lists notifiable infectious diseases [10]. The list has been constantly revised since it was first introduced; to date, 39 infectious diseases are listed due to their easy transmission, high prevalence, and severe consequences. Using epidemic levels and potential threats as basis, 39 notifiable infectious diseases are divided into three classes, i.e., classes A, B, and C (please find the list of 39 notifiable infectious diseases in S1 Table in supporting information), with severity decreasing across classes [11]. Class A (infectious diseases that require compulsory response measures) includes plague and cholera, which can cause large and rapid epidemics. However, only a few cases have been reported during the past decades. Class B (infectious diseases that require strict control and prevention measures) includes 26 diseases, which feature relatively strong infectivity, e.g., acquired immunodeficiency syndrome (AIDS), measles, and malaria. Class C (infectious diseases that require proper surveillance) includes less severe and endemic infectious diseases, such as influenza and typhus [12].

Even though many of the notifiable infectious diseases are active all over the country, people do not suffer equally as their incidents are not randomly distributed geographically. The spatial cluster feature is an inherent attribute of infectious diseases, providing an important basis for formulating prevention and control measures. As proposed by the WHO, the battle against infectious diseases should focus on geographic areas and populations that are at the highest risk [8]. Therefore, understanding the spatial distribution of infectious diseases and identifying the cluster areas are crucial. In the literature, initial efforts have been made to explore the spatial distribution of certain notifiable infectious diseases to support evidence-based policy making. For example, Fang and his colleagues [13] conducted a geographic information system (GIS)-based spatial analysis of hemorrhagic fever with renal syndrome (HFRS) in mainland China and detected the clusters of incidence at the county level. Zhang et al. [14] applied exploratory spatial data analysis to map out the spatial distribution patterns of incidence rates for human brucellosis throughout China. Using spatial autocorrelation statistic and space–time scan statistics as basis, Liu et al. [15] and Deng et al. [16] detected the spatial-temporal clusters of hand, foot, and mouth diseases (HFMD) in the Shandong and Guangdong Provinces, respectively. Hu et al. [17] compared the spatial clusters of schistosomiasis in southeast China between 1999–2001 and 2007–2008 to assess the progress of a World Bank Loan Project. Huang et al. did a spatial clustering analysis to explore the spatial epidemiology of pulmonary tuberculosis in the northeast of the Yunnan province [18]. Wu et al. mapped the county-level epidemiology and spatio-temporal distribution of HFMD incidence from 2009 to 2015 [19]. By contrast, Chen et al. [20] described and mapped the spatial clusters of incidence of schistosomiasis in the Hubei Province. Additional spatial analysis was conducted on notifiable infectious diseases, such as AIDS in the Yunnan [21] and Guangdong [22] Provinces, tuberculosis in the Zhejiang [23] and Qinghai Provinces [24], and malaria in Hubei Province [25].

Although knowledge has been accumulated, existing studies bear limitations. First, existing research, by and large, only focuses on one certain infectious disease. Up to now, only the spatial distribution patterns of a short list of notifiable infectious diseases are mapped out. Second, few research compared spatial distribution patterns or explored the connections of their clustering patterns, which limits the understanding of notifiable infectious diseases. The inter-correlations of the geographic clustering patterns of the listed epidemics can shed light on policy making for disease control. To fill the research gap, our study conducts a comprehensive analysis to cover the most common notifiable diseases and compare their spatial distribution patterns. We hope that this study can provide additional implications for making region-oriented, disease-specific, and multiple-disease-targeted disease control policies.

## Methodology

### Data collection

In this retrospective study, the provincial incidence data of notifiable infectious diseases in 2015 are obtained from the latest China Health and Family Planning Statistical Yearbook [26], which is published by the central government of China and is considered the country’s data authority. Hainan is not included in the spatial analysis part, considering the border requirement. Hong Kong and Macau are also excluded because of data accessibility.

It should be noted that not all notifiable infectious diseases are included in this study. Among the 39 notifiable infectious diseases identified by the *Law of the People’s Republic of China on the Prevention and Treatment of Infectious Diseases*, two class A diseases are almost eradicated, and the class C epidemics can only cause less severe consequences, thus making class B diseases a priority. Therefore, in this study, we only focus on class B epidemics. Considering the comparability of spatial distribution patterns, we also exclude diseases that are confined to certain regions. In other words, we only discuss 12 class B notifiable infectious diseases, which have reported cases in all the provincial units in mainland China. These 12 class B notifiable infectious diseases are viral hepatitis (hepatitis caused by different kinds of viruses is listed as a single disease in the law), dysentery, typhoid and paratyphoid (typhoid and paratyphoid are counted as a single disease type in the law), AIDS, gonorrhea, syphilis, measles, pertussis, scarlet fever, brucellosis, tuberculosis, and malaria. Please see the original incidence data of 12 class B notifiable infectious diseases in each provincial unit in S2 Table in supporting information.

### Procedures and spatial analysis

To fully understand the spatial distribution of notifiable infectious diseases, two forms of Moran’s I and corresponding graphic tools are adopted to explore and visualize the global and local spatial autocorrelation of incidence, i.e., pair-wise correlation of georeferenced observations for incidence rates [27]. Global Moran’s I is a measure describing the overall spatial distribution characteristics in an area as whole. Local Moran’s I, which is also referred to as a decomposition of global Moran’s I, is an indicator for a particular area, which can be used to detect the spatial clusters of infectious diseases [28]. Based on the incidence of one certain infectious diseases, we will calculate the global and local Moran’s I for each infectious disease and develop corresponding graphics. Then we can visually compare the various distribution patterns of 12 class B notifiable infectious diseases.

#### Spatial weight matrix

A spatial-weighted matrix (W), an n×n matrix containing the location information between each pair of target geographic units, serves as the premise for spatial autocorrelation analysis [29]. Taking the bordering standard as the gauge for measuring spatial weight has become a commonly adopted practice for researchers [30]. In our study, if geographic units *i* and *j* are adjacent, the corresponding value of *W*_*ij*_ is 1 and vice versa. However, if units *i* and *j* do not border each other, *W*_*ij*_ = 0. In our discussion, a geographic unit is a province or the provincial level of municipalities. We row-standardize the matrix to eliminate the influence of the number of neighbors.

$$W_{ij}=\left\{ \begin{array}{c} 1\;if\;provincial\;units\;i\;and\;j\;are\;bordering\;units\\ 0\;otherwise\; \end{array}\right.$$

#### Global Moran’s I statistic

Among all the indicators demonstrating geographic distribution of target observations, Moran’s I is universally adopted [31]. It directly indicates the clustering of similar or discrepant cases or a subset of cases, rendering it a powerful tool to explore the spatial autocorrelations between bordering units. The global Moran I, a figure ranging between −1 and 1, reveals the overall relationship of all the geographic units in the whole area of investigation. If global Moran’s I is positive, the value represents a positive spatial autocorrelation (cluster of similar values, high-high and low-low), whereas a negative value indicates the opposite (cluster of dissimilar values, high-low and low-high). Such tendency is more significant when the value approaches −1 and 1, whereas 0 embodies a random geographic distribution of all the units [32]. The calculation of global Moran’s I is shown in the following formula, in which *y*_*i*_ and *y*_*j*_ are the incidence rates of certain infectious disease of provincial units i and j, $\bar{y}$ is the average value. *W*_*ij*_, as explained in the previous paragraph, stands for the spatial-weighted matrix.

$$Global\;{Moran}^{\prime }s\;I=\frac{n\sum_{i=1}^{n}\sum_{j=1}^{n}W_{ij}(y_{i}-\bar{y})(y_{j}-\bar{y})}{\sum_{i=1}^{n}\sum_{j=1}^{n}W_{ij}\sum_{i=1}^{n}{\left(y_{i}-\bar{y}\right)}^{2}}$$

#### Moran scatterplots

The spatial distribution patterns of the unit values are illustrated in the Moran scatterplots, which is a direct visualization of the relationship between the incidence of each provincial unit and the weighted mean value of the bordering units. The horizontal axis stands for the incidence of each geographic unit, whereas the vertical one represents the lag (the weighted mean value of bordering units) of each plot [33]. Each of the 31 provincial units is represented by one plot in the diagrams, and global Moran’s I is reflected by the slope of the fitting line. To facilitate visualization, all the values are standardized.

A Moran scatterplot will be made for each kind of infectious diseases, in which the four quadrants split by the horizontal and vertical axes represent different spatial autocorrelation relationships. Quadrants I and III indicate a positive relationship (high–high and low–low, respectively), whereas II and IV reveal negative ones (high–low and low–high, respectively). For instance, units in quadrant I mean that their incidence value is above the mean and they are surrounded by units with values above average. Units in quadrant II show that a sub-average value is surrounded by values above average [34]. The same logic applies to quadrants III and IV as well. As a result, all provincial units are categorized into four groups in line with the spatial autocorrelation relationships.

#### Local Moran’s I statistic

When investigating the spatial autocorrelation of certain research targets on a large scale, different geographical units may reveal great disparities. Local Moran’s I, which is also known as a LISA (local indicator of spatial association), is a measure to describe the local spatial distribution characteristics [34]. Local Moran’s I is the result of disintegrating the global Moran’s I into each geographic area, with the sum of local Moran’s I being proportional to the global Moran’s I. It is defined by the following formula:

$${Local\;Moran}^{\prime }s\;I=\frac{\left(y_{i}-\bar{y}\right)}{m_{0}}\sum_{j}W_{ij}(y_{j}-\bar{y})\;m_{0}=\sum_{i}{\left(y_{i}-\bar{y}\right)}^{2}/n.$$

In the formula, “the operation of summing *j* is limited to the surrounding areas of *i*” as this measure only focus on the one certain area [30]. m_0_ is a consistent but not unbiases estimated of the variance, which is a constant for all locations [34]. *W*_*ij*_ is the spatial weight matrix, which is same with global Moran’s I. Similarly, local Moran’s I is a number between −1 and 1, with a positive number indicating clustering of similar values, whereas a negative number indicates the opposite. Proximity to −1 and 1 means a significant trend of such relationship, whereas approaching 0 indicates the opposite. Local Moran’s I can be used to identify the spatial clusters of infectious diseases [35]. In this study, the local Moran’s I is used to make the univariate LISA cluster maps.

#### Univariate LISA cluster map

Univariate LISA cluster maps are maps which highlight the spatial cluster areas. It is also connected with Moran scatterplots, the units that lie far from the origin of the coordinate in the scatterplots are deemed as outliers or leverage points, indicating that they are significant values among units in the same quadrant [34]. For such units, their LISA value is significant and if we visualize them on maps, we can obtain four types of spatial clusters (high-low, unit with high incidence surrounded by units with low incidence; high-high, unit with high incidence surrounded by units with high incidence; low-low, unit with low incidence surrounded by units with low incidence; low-high, unit with low incidence surrounded by units with high incidence) in accordance with the four quadrants of the diagram of the scatterplots. Monte Carlo randomization (99999 permutations) was employed to assess the significance of local Moran’s I, with the null hypothesis being that the distribution of one certain infectious disease in China is completely random distributed. To facilitate the understanding of readers, we display spatial clusters (i.e, units whose local Moran’s I reach a significance level of 0.05 or above) on China’s provincial map, which researchers also refer to as the “univariate LISA cluster map” [30]. Those units whose p-values are larger than 0.05 are classified as “not significant” on the maps. For cross reference, the incidence of each disease in each provincial unit, divided into four scales, is also illustrated in hierarchical map.

### Software tool

The global and local Moran’s I and Moran scatterplots are computed using GeoDa 1.8.16 [36]. Hierarchical maps, univariate LISA maps, and cluster frequency maps are developed with ArcGIS 10.0. Inter-correlation graphs of spatial cluster patterns are made with Circos-0.69–3 [37].

## Results

### Situation of infectious diseases in China

We list all the class B notifiable infectious diseases that are active in each provincial unit. Table 1 shows that the selected 12 diseases account for 98.33% of all reported class B infectious diseases in 2015, proving the representativeness of our selection.

**Table 1:** Description of the 12 class B notifiable infectious diseases (1/100000).

Notifiable infectious diseases	Class	Reported cases in 2015	Percentage of Class B cases	Incidence rate (1/100000)	Maximum incidence at provincial level	Minimum incidence at provincial level
**Gastrointestinal diseases**						
** Dysentery**	B	138917	4.56%	10.20	52.79	0.8
** Typhoid and paratyphoid**	B	11637	0.38%	0.85	6.93	0.03
**Sexually transmitted infections**						
** AIDS**	B	50330	1.65%	3.69	13.25	0.62
** Gonorrhea**	B	100245	3.29%	7.36	29.82	1.72
** Syphilis**	B	433974	14.25%	31.85	107.51	13.5
**Vaccine-preventable diseases**						
** Measles**	B	42361	1.39%	3.11	31.96	0.16
** Pertussis**	B	6658	0.22%	0.49	5.63	0.00
**Bacterial infections**						
** Scarlet fever**	B	68249	2.24%	5.01	19.29	0.14
** Tuberculosis**	C → B (2004)	864015	28.36%	63.42	43.66	0.01
**Zoonotic and vector-borne diseases**						
** Brucellosis**	B	56989	1.87%	4.18	184.53	19.52
** Malaria**	B	3116	0.10%	0.23	1.09	0.01
**Viral hepatitis**	B	1218946	40.01%	89.47	233.58	13.82
** Total**	–	2995437	98.33%	219.86	–	–

Table 1 is a descriptive summary of the 12 selected notifiable epidemics in China in 2015. A universal taxonomy is applied in our study to sort the disease into 6 clusters. They are gastrointestinal diseases (dysentery and typhoid and paratyphoid), sexually transmitted infections (AIDS, gonorrhea, and syphilis), vaccine-preventable diseases (measles and pertussis), bacterial infections (scarlet fever and tuberculosis), zoonotic and vector-borne diseases (brucellosis and malaria) as well as the different types of viral hepatitis. Along with the regular profile of reported cases and incidence of each disease (per 100,000 people), Table 1 also presents the maximum and minimum incidences of each disease at provincial level.

Viral hepatitis ranks first and takes 40.01% of class B epidemics, with 1,218,946 reported cases in 2015. Tuberculosis is second to viral hepatitis, which has 864,015 reported cases (28.36%). The third most common infectious disease is syphilis, whose share and number of cases are 14.25% and 433,974 respectively. That all the other selected epidemics are less prominent regarding their number of reported cases, incidence per 100,000 populations, and overall share is worth mentioning. For instance, the incidences of dysentery, gonorrhea, scarlet fever, brucellosis, AIDS, and measles ranges from 5% to 1%, with the values decreasing in turn. The rest of the epidemics, namely typhoid and paratyphoid, pertussis, and malaria, have a share lower than 1%, whose reported cases are 11637, 6658, and 3116, respectively.

### Global spatial autocorrelation

**Fig 1:**
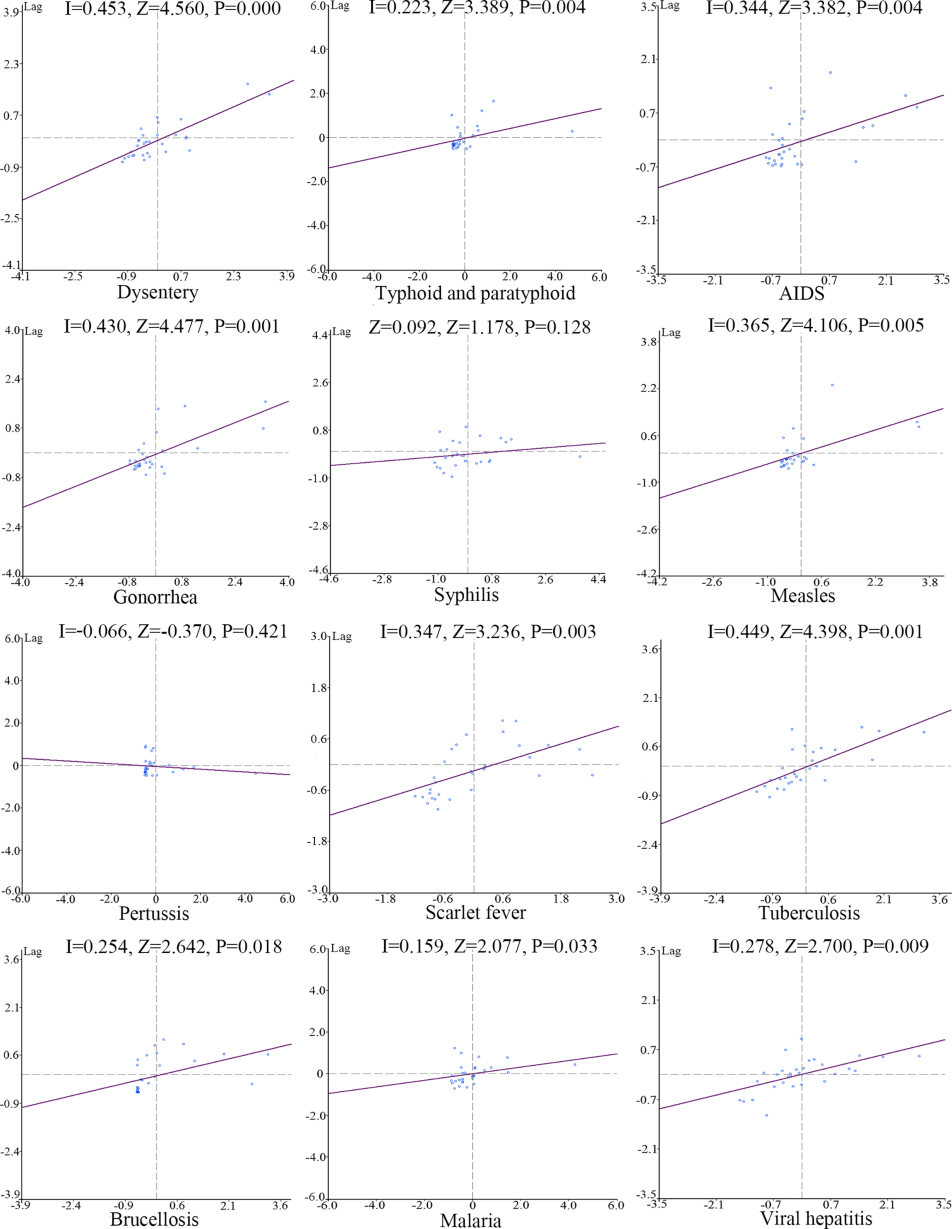
Moran scatterplots of the incidence rates of the 12 notifiable infectious diseases in China.

Fig 1 illustrates the Moran scatterplots of the 12 diseases. Moran’s I values are represented by the slope of the fitting lines. A greater slope means a greater value of Moran’s I, suggesting a more unevenly distribution of the incidence of the epidemics throughout the country. Each plot in the diagrams stands for a provincial unit. As explained in the method, the horizontal and vertical axes split each diagram into four quadrants. The plots in quadrants I and III represent the high–high and low–low distribution patterns of the provincial units, whereas quadrants II and IV stand for a high–low and low–high distribution pattern.

Overall, almost all the 12 class diseases, except for syphilis and pertussis, display a significant positive global spatial autocorrelation (P<0.05). Dysentery, tuberculosis, and gonorrhea feature the most unbalanced distribution among all the 31 provincial units. The three diseases have global Moran’s I values of 0.453, 0.449, and 0.430, respectively. Equally important is that, in terms of the three diseases, the majority of the provincial units fall in the third quadrant, yet they do have units lie far above the first quadrant. The uneven distribution of incidence of measles (global Moran’s I = 0.365), AIDS (0.344), and scarlet fever (0.347) among the provincial units is also significant, with their Moran’s I values ranging from 0.3 to 0.4. However, their plot distribution pattern is quite different. For the former two, the provincial units are mainly concentrated in quadrant III, despite a number of units being far from the origin of the coordinator in quadrant I. As for the latter, the provincial units have a more balanced distribution in quadrants I and III. All the other diseases either have a lower positive/negative value of Moran’s I, indicating that the spatial autocorrelation is not obvious. Besides, as different types of viral hepatitis differ in transmission route and physical consequences, we also developed the Moran scatterplots for different types of viral hepatitis (A, B, C, E) in S1 Fig in supporting information. Nevertheless, the diagrams of scatterplots only reveal the extent to which the diseases are unevenly distributed in the provincial units in general. More details about the local spatial distribution characteristics are needed.

### Local spatial autocorrelation

**Fig 2:**
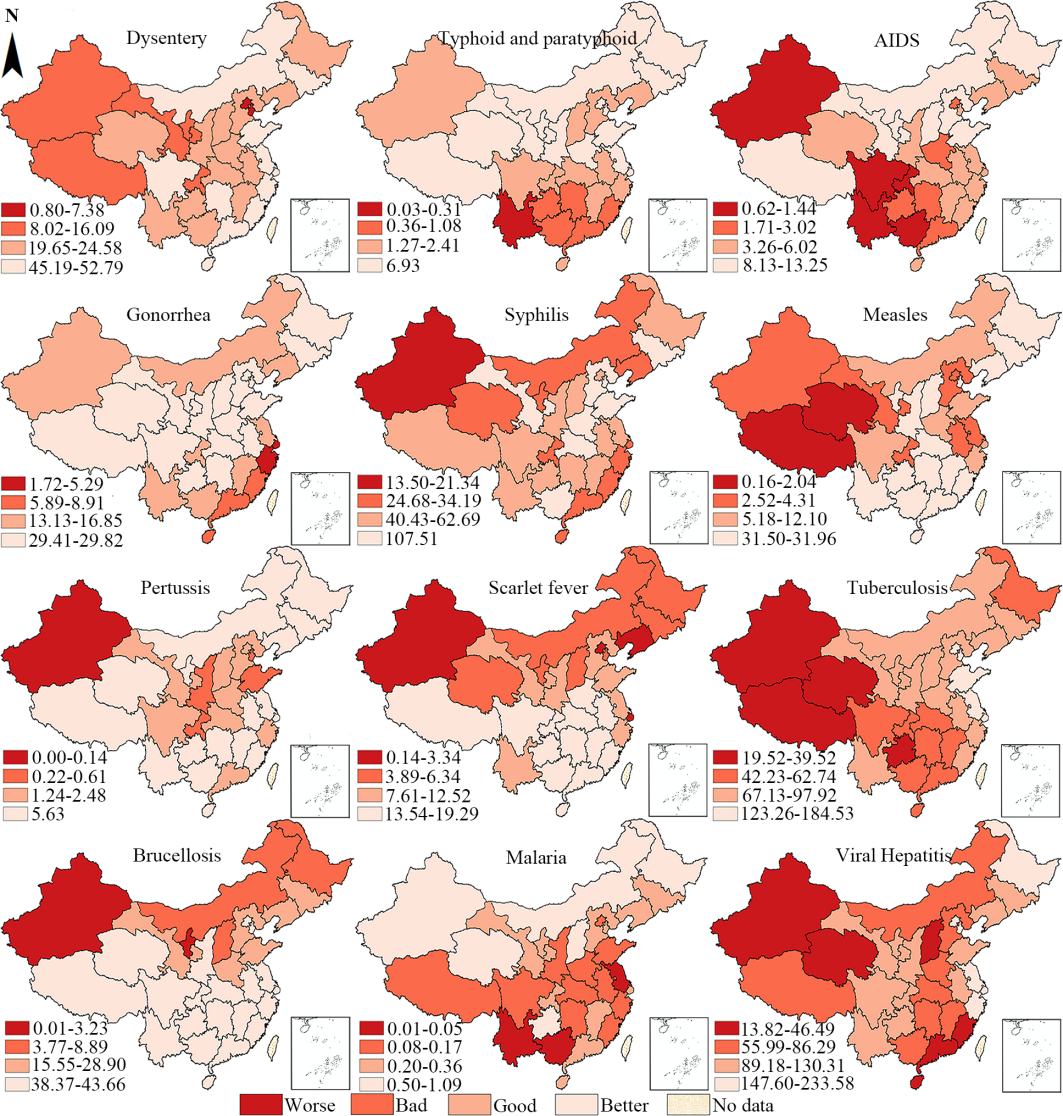
Hierarchical map of the incidence rates of the 12 notifiable infectious diseases in China (Unit: 1/100000).

Fig 2 presents a hierarchical map of the incidence per 10,000 populations of the 12 notifiable infectious diseases in the 31 provincial units, the results for different types of viral hepatitis (A, B, C, E) are displayed in S2 Fig in supporting information. Four scales, indicated by worse (dark red) to better (light red), are created with the natural break method. It is a commonly adopted scaling method to maximize the variance between scales while minimize it within the scales [38]. The hierarchical map is a very basic geographic summary of the 12 epidemics. For example, scarlet fever and brucellosis are more severe in the northern provinces, whereas malaria, AIDS, and typhoid and paratyphoid are more pandemic in the southern provinces. Other diseases, such as tuberculosis, are more concentrated in the west and southwest provinces.

**Fig 3:**
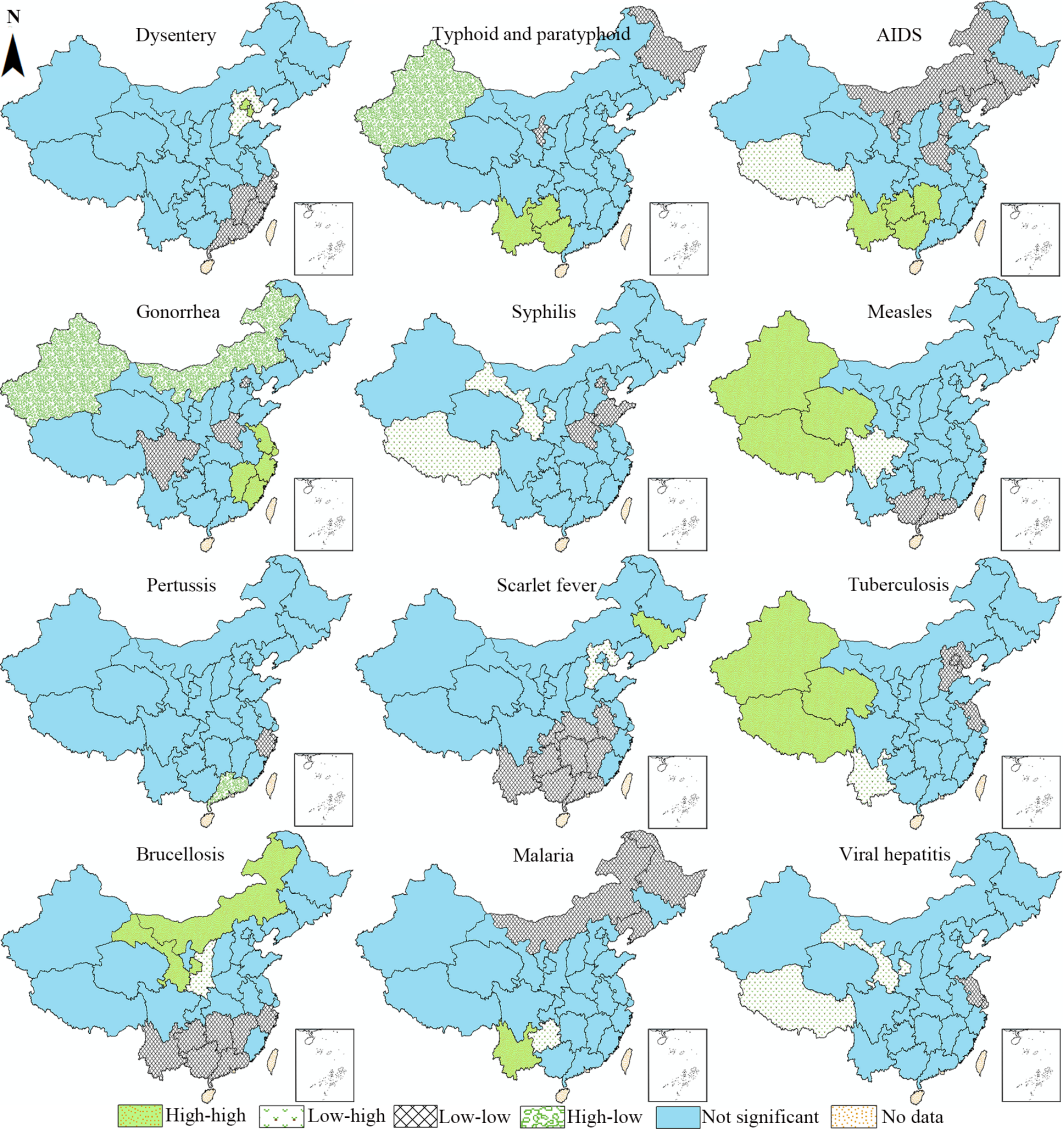
Univariate LISA cluster map of the incidence rates of the 12 notifiable infectious diseases in China.

The spatial cluster areas detected by local Moran’s I are visualized in the univariate LISA maps in Fig 3. As the incidence of these infectious diseases displayed positive autocorrelation, and therefore we could find more high-high (HH) and low-low (LL) cluster areas on the maps. It is easy to conclude that every selected infectious disease reveals a unique spatial distribution pattern, while some diseases still share similarities in the spatial clusters. Regarding the HH cluster areas, three western provincial units with large territories (Xinjiang, Xizang, and Qinghai) are in the HH clusters of tuberculosis and measles. AIDS and typhoid and paratyphoid also share similar geographical HH clusters with the former encompassing four southwest provinces (Guangxi, Yunnan, Guizhou, and Hunan) and the latter encompassing three provinces (Guangxi, Yunnan, and Guizhou). As for the LL cluster areas, the four southeast provinces (Jiangxi, Zhejiang, Fujian, and Guangdong) constitute a LL cluster regarding the incidence of dysentery. Similarly, brucellosis and scarlet fever overlap in a vast area in southern China in terms of the LL clustering pattern, suggesting that the diseases are less pandemic in the southern provinces.

### Comparison of the spatial distribution patterns

To further compare the varying distribution patterns of 12 selected class B epidemics, it is essential to compare and summarize the frequency of spatial cluster features by disease and location. Based on the high or low value in local units, we divide the four cluster types into two categories, i.e., high type (high–high and high–low) and low type (low–low type and low–high) of clusters. The former identify units with values higher than the mean, whereas the latter specify the ones with lower values.

**Fig 4:**
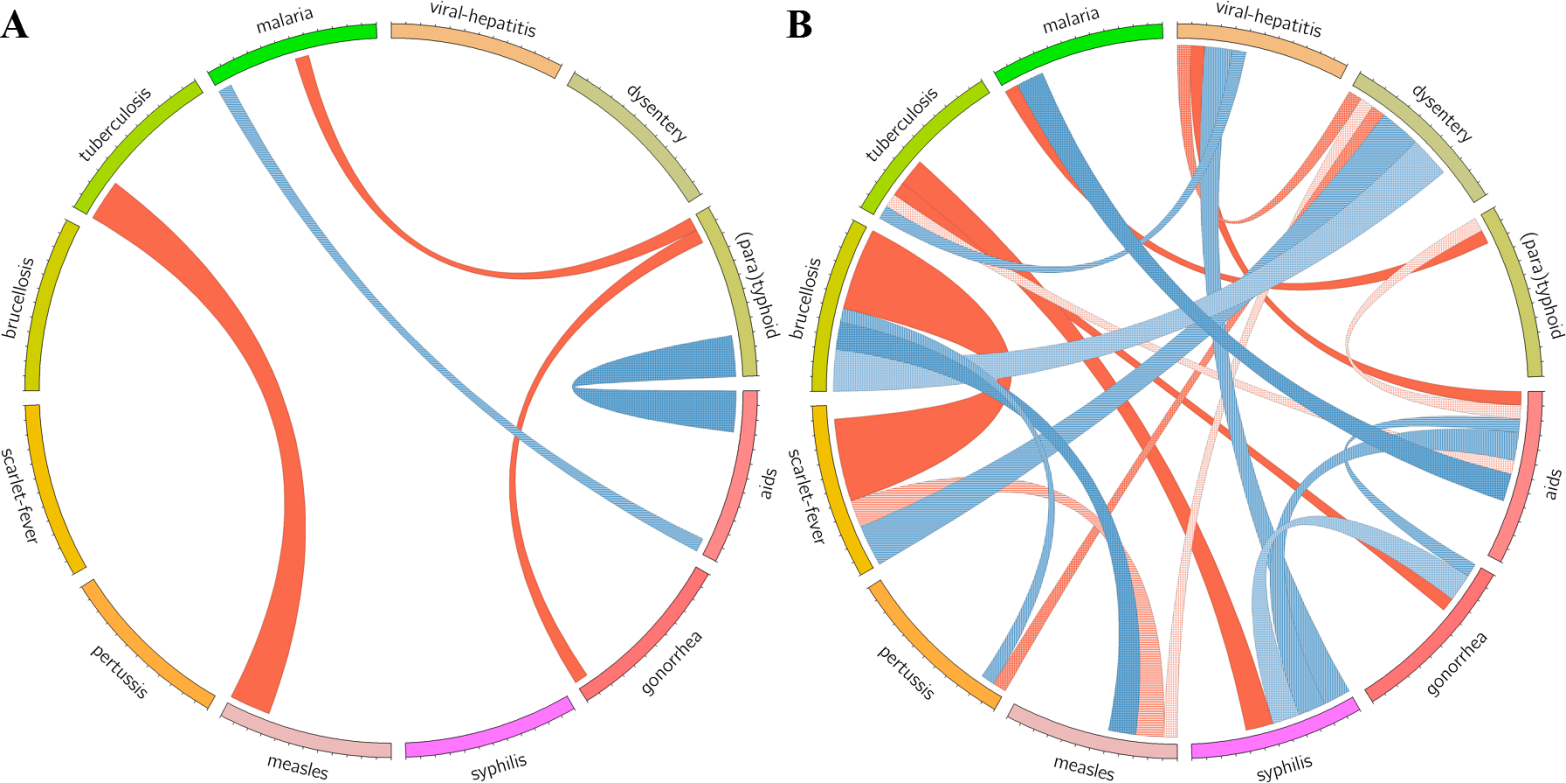
Inter-correlations of the clustering patterns of every two diseases (A for high–high and high–low clusters and B for low–low and low–high clusters).

Fig 4 shows the inter-correlations of the clustering patterns between every two notifiable infectious diseases. Fig 4A only displays the high–high and high–low types, whereas Fig 4B displays the remaining two types. The link indicates the co-occurrence of the same type of spatial cluster between two infectious diseases. The widths of the “ribbons” in the pictures represent the frequency of links, the wider the “ribbons,” the higher the frequency. For instance, the incidence rate of both malaria and AIDS displays a significantly HH cluster feature in Yunnan, then the width of the “ribbon” connecting AIDS and malaria in Fig 4A is 1. In addition, the color of the “ribbons” was randomly generated to better distinguish the “ribbons”. As shown in Fig 4A, the links for the high-type is simple in the figure, with only five links, i.e., measles and tuberculosis (3), malaria and AIDS (1), malaria and typhoid (1), typhoid and AIDS (3), and typhoid and gonorrhea (1). Whereas the links of the low-type of spatial clusters (Fig 4B) are much more complicated, all infectious diseases co-occur the same type of spatial cluster in certain units with others. The maximum number of occurrences is between brucellosis and scarlet-fever (6).

**Fig 5:**
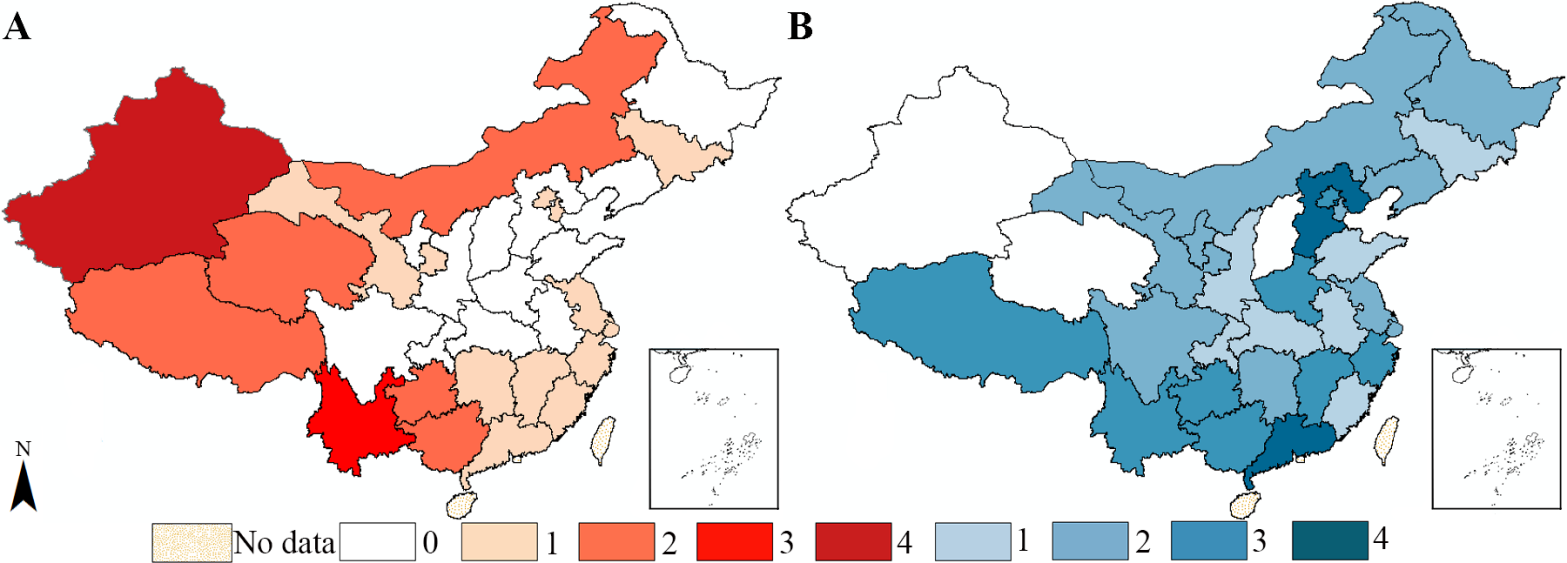
Frequency of cluster occurrence among the 12 notifiable infectious diseases (A for high–high and high–low clusters and B for low–low and low–high clusters).

Fig 5 shows the frequency of cluster occurrence of the 12 notifiable infectious diseases. The high type of spatial clusters (high–high and high–low) tends to be found in west China. The maximum number of occurrences is found in Xinjiang (4) and Yunnan (3), and the provincial units without the high type of clusters are concentrated in north and east China. The spatial distribution of low–low and low–high clusters is exactly the opposite, many low–low and low–high types of cluster occurs in east China, with Hebei (4) and Guangdong (4) ranking first.

## Discussion

To strengthen China’s capacity to defend against notifiable infectious diseases, we need to investigate their spatial distribution patterns. In this study, we performed spatial analysis of 12 class B notifiable infectious diseases, summarized, and compared their spatial distribution patterns. Although not all class B infectious diseases are included in this study, 12 targeted notifiable infectious diseases, which account for 98.33% of all reported class B cases and are active all over the country. It justifies the representativeness and generalizability of the study. The findings provide comprehensive understanding of the epidemics in China and serve as a basis for effective regional policies to prevent and control notifiable infectious diseases.

The incidence rates of almost all the 12 class B notifiable infectious diseases, except pertussis, exhibit positive spatial autocorrelation because notifiable infectious diseases are highly contagious. This finding echoes that of previous studies on one single infectious disease in one provincial unit [14,24,25], although the correspondence between values of global Moran’s I and spatial autocorrelation intensity is not defined. Compared with the values of global Moran’s I for other issues which display positive spatial autocorrelation and arouse regional cooperation in China, such as 0.234 for carbon activity in 2012 [31], and 0.325 for PM_2.5_ (main component of air pollution, particulate matter with a diameter of 2.5 μm or less) in 73 cities in 2013 [39], global Moran’s I for many infectious diseases such as dysentery, syphilis, and tuberculosis, are even higher than 0.4, which is an indication of strong spatial autocorrelation. The results of global Moran’s I verify the fact that that no provincial units can contain the epidemics without cooperating with other provinces in the war against infectious diseases. No area can shield itself from the consequences of an infectious disease crisis.

Regarding the detailed spatial distribution of the 12 notifiable infectious diseases, all diseases have differing geographical concentrations. To facilitate the comparison among different diseases, we discuss the spatial clusters by disease categories. First, viral hepatitis was relatively neglected during the MDG era [8]. The number of reported cases in 2015 accounts for 40.01% of all the 12 diseases. As different types of viral hepatitis are considered as one on the notifiable infectious disease list, we map out their spatial distribution as a sum and also the distribution patterns of its genotypes (hepatitis A, B, C, E) in the appendix. Based on their relatively different spatial distribution patterns, it is essential to formulate the specialized prevention and control measures for different types of viral hepatitis, especially for hepatitis B [11]. Second, among sexually transmitted diseases (AIDS, gonorrhea, syphilis), the values of global Moran’s I for AIDS and gonorrhea are higher than 0.3, indicating that it is more challenging and less optimistic for the prevention and control of these two diseases. According to the trend analysis of Zhang and Wilson [12], the incidence rates of these kinds of diseases have continued to rise since the 1990s. It is important to note that in 1985, the first case of AIDS in China was discovered in Yunnan province, which is situated in the southwest border of China [21]. Up to now, provinces (Yunnan, Guizhou, Guangxi and Hunan) in southwest and mid-south China is still suffering from AIDS as the high–high cluster areas all concentrate in these areas. HH cluster areas for gonorrhea are located in the south-east areas (Jiangsu, Shanghai, Zhejiang, Fujian and Jiangxi). In contrast, even though the incidence of syphilis is much higher, no significant high–high cluster areas are identified in the space, which means that the incidence of syphilis is almost evenly distributed in the space.

Third, vaccines are powerful weapons in the human arsenal against infectious diseases [40]. Measles and pertussis are typical vaccine-controlled diseases in China. Comparatively speaking, the control of pertussis is more successful as the incidence of pertussis is significantly lower than that of measles. In addition, no positive autocorrelation is noted for the incidence of pertussis, indicating that pertussis has been effectively controlled. In contrast, HH cluster feature of measles is detected in three western provinces (Xinjiang, Xizang and Qinghai). The difference between their incidence rates may lies in the low coverage of the second dose of measles vaccine in China despite a recommendation of two-dose vaccines [11,41]. In addition, the medical facilities and manpower in the western part of China is an inferior position compared with other regions.

Fourth, the incidence of infectious gastrointestinal diseases in China displays a downward trend according to Zhang, Wilson [11] and Wang et al. [42]. However, dysentery and typhoid (typhoid and paratyphoid) remain active all over the country. Comparatively, dysentery features higher prevalence and stronger positive spatial autocorrelation in norther provincial units of Beijing and Tianjin, while provinces (Yunnan, Guangxi, Guzizhou) in the southwest are cluster areas that demand attention to combat the typhoid and paratyphoid. Regarding zoonotic and vector-borne diseases, the incidence rate of malaria is the lowest among the 12 diseases in this study, even though there are also reported cases of Malaria in each provincial unit. Only the high–high cluster in Yunnan province reached a significant level. Although the incidence of brucellosis is relatively higher, Neimenggu and Gansu are the only provincial units displaying a high–high cluster.

Finally, regarding bacterial infections (scarlet fever and tuberculosis), the incidence of tuberculosis ranks much higher (2nd) among the 12 diseases, with Xinjiang, Xizang, and Qinghai identified as high–high cluster areas. In recent years, China reveals not only a high incidence of tuberculosis but also a serious epidemic of drug-resistant tuberculosis. According to the national survey of drug-resistant tuberculosis in China, all provinces surveyed in China had different levels of multidrug-resistant (MDR) tuberculosis[43]. Certain provinces are even classified as global cluster areas of MDR tuberculosis, which poses a conundrum for tuberculosis control in China.

Despite the various spatial clusters of the 12 notifiable infectious diseases, more similarities and differences can be noted if we compare and summarize their spatial cluster areas. The provincial units which display low–low or low–high cluster features are most likely to be located in the northern and eastern provincial units. In contrast, despite the scarce population in west provinces which may, to a certain extent, limit the spread of infectious diseases, more high–high and high–low cluster types are situated in this region, indicating a much more serious condition in less-developed areas. Regarding the inter-correlation of the clustering patterns of diseases, certain infectious diseases display very similar clustering patterns, which provides new evidence for disease control in China. For instance, Xinjiang, Xizang, and Qinghai display high–high cluster type for measles and tuberculosis, therefore the government may launch a special project for these diseases in these provincial units.

Being an ecological study, this study faces the risk of ecological fallacy. A higher incidence in one provincial unit does not mean a higher risk to get infected for people living in this unit, while the incidence is the most widely used indicator to represent the situation and the basis for disease prevention and control. We believe that the results have certain value even though it is a macro-level study. Besides, this study only focuses on the spatial distribution of infectious diseases, thus limiting its policy implications. As a result, different units should take into account the specific economic and social conditions when formulating corresponding policies.

## Conclusions

In 1970, the Surgeon-General of the United States of America indicated that it was "time to close the book on infectious diseases, declare the war against pestilence won, and shift national resources to such chronic problems as cancer and heart disease" [44]. However, the emerging spatial techniques have proven that, the further we go, the harder it becomes. Many traditional infections have not been effectively controlled, whereas emerging infectious diseases take place one after the other. Attention paid to and action against infectious diseases can never be lax unless all infectious diseases are under effective control.

An improved understanding of the spatial distribution of incidence rates of infectious diseases is a starting point in identifying diseases and areas which need targeted public health interventions. The next step is to use these findings to formulate evidence-based, disease-specific, location-specific and disease-combined interventions that will promote the prevention and control of infectious diseases in China. Various concentrations of notifiable infectious diseases indicate a severe situation in China. The clustering patterns of infectious diseases proffer new perspectives to epidemic control and intervention. On one hand, coordinated intervention and control methods across the borders of adjacent provinces are necessary for diseases that have a particular regional concentration. It will be less effective, if not futile for the government to combat a certain infectious disease within one provincial unit when it borders with other HH clustering provinces. For example, AIDS is particularly serious in Yunnan, Guizhou, Guangxi and Hunan. The prevention and control mechanisms will be substantially muted without inter-provincial coordination at a time of free and constant population mobility. The same logic applies to other diseases (typhoid and paratyphoid, measles, gonorrhea, tuberculosis and brucellosis) where across-scale and inter-region cooperation is urgent. Besides, different provincial units need to adopt distinctive control and prevention methods regarding the geographical characteristics of a certain disease. For example, in the HL cluster areas, the bordering units need to prevent the spread of the disease while in the HH cluster areas, mobility control of diseases among the bordering units will be the key issue.

On the other hand, for diseases that share similar geographical clustering patterns, a comprehensive reaction package targeting multiple diseases is crucial to improve the efficiency and effectiveness of policies. For example, for the health authorities in Xinjiang, Xizang and Qinghai, a package of control and prevention methods need to be contrived to deal with measles and tuberculosis at the same time. The similarity in the geographical distribution across different diseases may indicate shared inadequacy in medical resources provision and negligence in supervising & inspecting mechanisms. A policy package targeting at various diseases will dramatically increase the effectiveness and efficiency of current diseases control mechanisms. In addition, in the SDG (Sustainable Development Goal) era, infectious disease prevention is not the sole responsibility of the health sector because the spread of infectious diseases is affected by multiple socioeconomic, environmental, and ecological factors, as well as rapidly increasing antimicrobial resistance. Increased cross-sector cooperation is needed to better prevent and control the spread of infectious diseases. However, for brevity, we do not include other minor infectious diseases. We suggest further research on this issue, digging into the spatial autocorrelation and inter-connections of all infectious diseases.

## Supporting information

S1 Table Classification of notifiable infectious diseases in China. (DOCX)

S2 Table Incidence data of 12 notifiable infectious diseases at the provincial level in 2015 (1/100000). (DOCX)

S1 Fig Moran scatterplots of the incidence rates of the hepatitis A, B, C, E in China.
(TIF)

S2 Fig Hierarchical maps and univariate LISA cluster maps of the incidence rates of hepatitis A, B, C, E in China.
(TIF)
