# Supplementary material for: Spatial distribution of 12 class B notifiable infectious diseases in China: A retrospective study
Source: PLoS One. 2018 Apr 5;13(4):e0195568. doi: 10.1371/journal.pone.0195568 (PMC5886686; doi:10.1371/journal.pone.0195568)
Supplement: S1 Table — (DOCX) [file pone.0195568.s001.docx]

S1 Table Classification of Notifiable Infectious Diseases in China

| Class | Items |
| --- | --- |
| Class A | Plague, Cholera |
| Class B | Severe Acute Respiratory Syndrome (SARS), Acquired immunodeficiency syndrome(AIDS), Viral hepatitis, Poliomyelitis, Human infections of highly pathogenic avian influenza, human infections of H7N9 avian influenza, Measles, Epidemic hemorrhagic fever(EHF), Rabies, Epidemic Encephalitis B, Dengue, Anthrax, Bacterial and amoebic dysentery, Tuberculosis, Typhoid & paratyphoid , Epidemic (meningococcal) meningitis, Pertussis, Diphtheria, Neonatal tetanus, Scarlet fever, Brucellosis, Gonorrhea, Syphilis, Leptospirosis, Schistosomiasis, Malaria |
| Class C | Influenza, Mumps, Rubella, Acute hemorrhagic conjunctivitis(AHC), Leprosy, Typhus, Leishmaniasis, Echinococciosis, Filariasis, Other infectious diarrheal diseases, Hand, foot and mouth disease(HFMD) |
