# Supplementary material for: Spatial distribution of 12 class B notifiable infectious diseases in China: A retrospective study
Source: PLoS One. 2018 Apr 5;13(4):e0195568. doi: 10.1371/journal.pone.0195568 (PMC5886686; doi:10.1371/journal.pone.0195568)
Supplement: S2 Table — (DOCX) [file pone.0195568.s002.docx]

S2 Table Incidence data of 12 Class B notifiable infectious diseases at the provincial level in 2015 (1/100000)

| Region | Viral hepatitis | dysentery | Typhoid and paratyphoid | AIDS | Gonorrhea | Syphilis | Measles | Pertussis | Scarlet fever | Brucellosis | Tuberculosis | Malaria |
| --- | --- | --- | --- | --- | --- | --- | --- | --- | --- | --- | --- | --- |
| Beijing | 13.82 | 45.19 | 0.14 | 3.61 | 5.14 | 24.68 | 6.14 | 0.43 | 17.94 | 1.31 | 31.97 | 0.21 |
| Tianjin | 18.12 | 52.79 | 0.13 | 1.79 | 2.7 | 19.37 | 1.78 | 2.48 | 11.06 | 1.75 | 19.52 | 0.11 |
| Hebei | 89.39 | 13.04 | 0.4 | 0.92 | 1.72 | 13.5 | 5.18 | 0.48 | 5.68 | 7.48 | 46.11 | 0.08 |
| Shanxi | 154.86 | 9.12 | 1.08 | 1.35 | 3.72 | 26.9 | 1.47 | 0.3 | 7.67 | 19.18 | 42.32 | 0.05 |
| Neimenggu | 117.36 | 6.61 | 0.1 | 0.83 | 8.33 | 43.41 | 2.89 | 0.06 | 11.4 | 28.9 | 47.71 | 0.02 |
| Liaoning | 75.19 | 9.69 | 0.38 | 1.88 | 5.89 | 40.43 | 0.41 | 0.01 | 14.55 | 6.65 | 53.72 | 0.15 |
| Jilin | 66.82 | 3.74 | 0.07 | 1.96 | 5.29 | 21.34 | 0.69 | 0.00 | 9.61 | 5.9 | 56.45 | 0.08 |
| Heilongjiang | 43.13 | 8.52 | 0.1 | 1.44 | 4.38 | 25.31 | 1.06 | 0.1 | 9.67 | 15.55 | 86.73 | 0.04 |
| Shanghai | 46.49 | 0.8 | 0.12 | 2.15 | 29.82 | 56.13 | 4.31 | 0.02 | 19.29 | 0.01 | 27.56 | 0.1 |
| Jiangsu | 29.55 | 4.9 | 0.2 | 1.98 | 7.65 | 29.64 | 5.69 | 0.04 | 4.1 | 0.1 | 39.52 | 0.51 |
| Zhejiang | 34.94 | 4.32 | 0.7 | 3.02 | 29.41 | 59.36 | 2.52 | 0.38 | 6.34 | 0.18 | 50.8 | 0.3 |
| Anhui | 79.16 | 16.09 | 0.49 | 1.71 | 4.64 | 34.19 | 5.48 | 0.04 | 1.77 | 0.12 | 58.44 | 0.22 |
| Fujian | 147.6 | 1.52 | 1.27 | 2 | 13.13 | 62.96 | 1.11 | 0.03 | 1.73 | 0.27 | 44.82 | 0.2 |
| Jiangxi | 98.27 | 10.37 | 0.51 | 2.6 | 7.27 | 25.11 | 0.39 | 0.04 | 0.15 | 0.1 | 71.63 | 0.13 |
| Shandong | 55.99 | 6.44 | 0.07 | 0.62 | 3.85 | 15 | 3.04 | 1.43 | 6.18 | 3.77 | 33.06 | 0.22 |
| Henan | 91.35 | 14.84 | 0.31 | 3.26 | 2.92 | 17.04 | 3.38 | 0.24 | 2.21 | 5.91 | 62.74 | 0.2 |
| Hubei | 130.31 | 8.02 | 0.47 | 2.02 | 3.87 | 20.67 | 1.75 | 0.22 | 1.97 | 0.44 | 78.14 | 0.21 |
| Hunan | 105.11 | 6.07 | 1.53 | 3.82 | 3.56 | 31.04 | 1.73 | 0.14 | 1.45 | 0.14 | 83 | 0.21 |
| Guangdong | 160.46 | 3 | 1.48 | 3.65 | 15.84 | 46.64 | 2.04 | 0.61 | 2.6 | 0.45 | 74.12 | 0.1 |
| Guangxi | 114.52 | 8.1 | 2.41 | 13.25 | 8.56 | 17.27 | 0.3 | 0.01 | 0.94 | 0.08 | 96.41 | 0.5 |
| Hainan | 171.3 | 4.5 | 0.38 | 2.1 | 16.85 | 48.59 | 0.16 | — | 0.14 | 0.03 | 97.92 | 0.17 |
| Chongqing | 86.29 | 24.58 | 0.5 | 8.76 | 5.93 | 48.55 | 7.88 | 1.24 | 2.73 | 0.16 | 75 | 0.11 |
| Sichuan | 65.38 | 7.38 | 0.36 | 9.55 | 3.37 | 27.83 | 3.13 | 0.36 | 2.36 | 0.05 | 67.13 | 0.36 |
| Guizhou | 75.93 | 8.4 | 1.74 | 6.02 | 4.76 | 32.37 | 0.7 | 0.12 | 2.28 | 0.19 | 133.46 | 0.04 |
| Yunnan | 72.88 | 10.27 | 6.93 | 12.31 | 6.42 | 33.02 | 0.86 | 0.06 | 3.89 | 0.46 | 54.42 | 1.09 |
| Xizang | 89.18 | 23.3 | 0.03 | 1.04 | 1.73 | 33.57 | 31.96 | 0.03 | 3.34 | 0.03 | 140.2 | 0.25 |
| Shaanxi | 82.94 | 13.15 | 0.1 | 1.74 | 3.47 | 24.91 | 1.64 | 1.96 | 6.18 | 3.23 | 56.66 | 0.2 |
| Gansu | 69.44 | 23.38 | 0.15 | 1.2 | 2.94 | 16.78 | 5.44 | 0.34 | 4.61 | 8.89 | 54.92 | 0.11 |
| Qinghai | 188.83 | 13.44 | 0.14 | 2.59 | 2.86 | 42.66 | 31.5 | 0.05 | 7.61 | 0.27 | 123.26 | 0.02 |
| Ningxia | 59.21 | 19.65 | 0.27 | 1.18 | 5.09 | 49.19 | 1.6 | 0.05 | 12.52 | 43.66 | 42.23 | 0.11 |
| Xinjiang | 233.58 | 21.55 | 0.87 | 8.13 | 8.91 | 107.51 | 12.1 | 5.63 | 13.54 | 38.37 | 184.53 | 0.01 |
| Total | 89.47 | 10.2 | 0.85 | 3.69 | 7.36 | 31.85 | 3.11 | 0.49 | 5.01 | 4.18 | 63.42 | 0.23 |

*Even though there is no reported cases of pertussis in Hainan, but as Hainan is not included in the spatial analysis, therefore the pertussis is also included in this study.
