# Supplementary figures and images for: Spatial distribution of 12 class B notifiable infectious diseases in China: A retrospective study
Source: PLoS One. 2018 Apr 5;13(4):e0195568. doi: 10.1371/journal.pone.0195568 (PMC5886686; doi:10.1371/journal.pone.0195568)

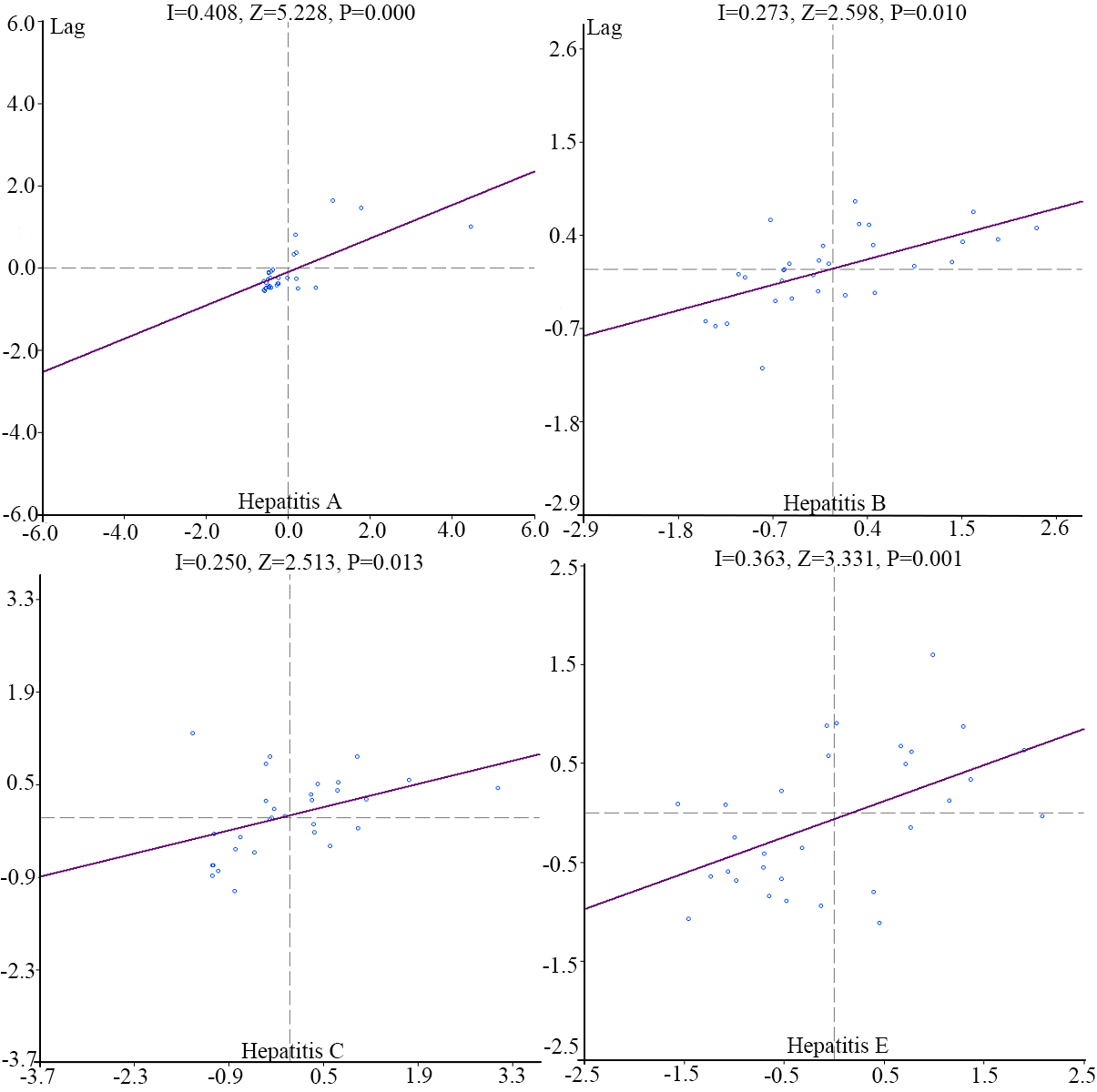

Supplement: S1 Fig — Moran scatterplots of the incidence rates of the hepatitis A, B, C, E in China. (TIF) [file pone.0195568.s003.tif]

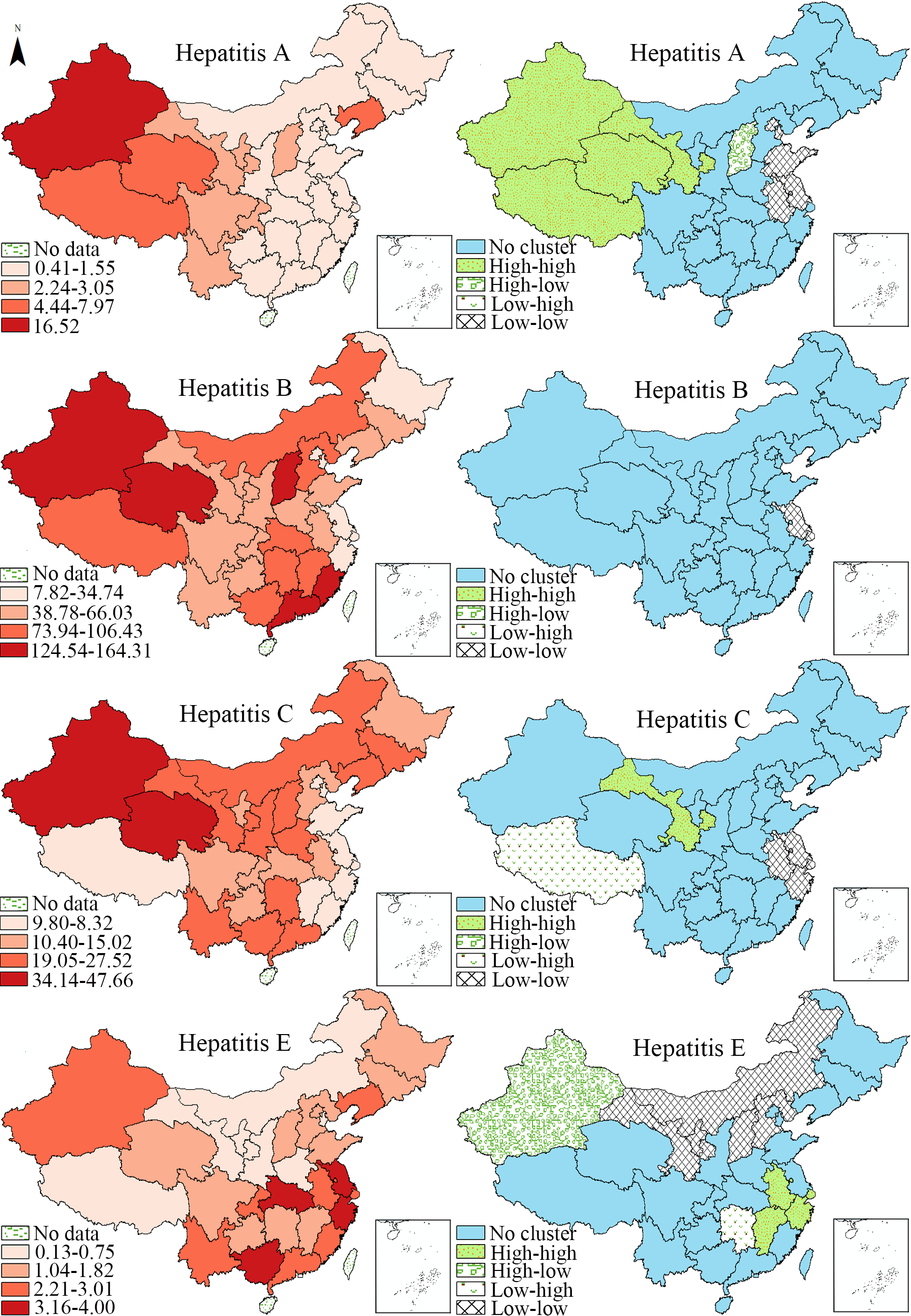

Supplement: S2 Fig — Hierarchical maps and univariate LISA cluster maps of the incidence rates of hepatitis A, B, C, E in China. (TIF) [file pone.0195568.s004.tif]
